# P2X7 Receptor Agonist 2′(3′)-O-(4-Benzoylbenzoyl)ATP Differently Modulates Cell Viability and Corticostriatal Synaptic Transmission in Experimental Models of Huntington’s Disease

**DOI:** 10.3389/fphar.2020.633861

**Published:** 2021-02-19

**Authors:** Alberto Martire, Rita Pepponi, Francesco Liguori, Cinzia Volonté, Patrizia Popoli

**Affiliations:** ^1^ National Center for Drug Research and Evaluation, Istituto Superiore di Sanità, Rome, Italy; ^2^ Preclinical Neuroscience, IRCCS Santa Lucia Foundation, Rome, Italy; ^3^ Institute for Systems Analysis and Computer Science “A. Ruberti”, National Research Council (IASI-CNR), Rome, Italy

**Keywords:** Huntington’s disease, P2X7 receptor, BzATP, OxATP, corticostriatal slices, adenosine A1 receptor, adenosine, DPCPX

## Abstract

Huntington’s disease (HD) is a life-threatening neurodegenerative disorder. Altered levels and functions of the purinergic ionotropic P2X7 receptors (P2X7Rs) have been found in animal and cellular models of HD, suggesting their possible role in the pathogenesis of the disease; accordingly, the therapeutic potential of P2X7R antagonists in HD has been proposed. Here we further investigated the effects of P2X7R ligands in *in vitro* and *ex vivo* HD experimental models. In ST14A/Q120 rat striatal cells, we found a reduction of P2X7R expression; however, the P2X7R agonist 2′(3′)-O-(4-benzoylbenzoyl)adenosine-5′-triphosphate (BzATP) induced cellular death, and this effect was fully reversed by the antagonist periodate-oxidized adenosine 5′-triphosphate (OxATP). Moreover, in corticostriatal slices from symptomatic R6/2 mice, BzATP reduced the synaptic transmission to a larger extent than in wild-type (WT) mice. Such an effect was accompanied by a concomitant increase of the paired-pulse ratio, suggesting a presynaptic inhibitory action. This was confirmed to be the case, since while the effects of BzATP were unaffected by the P2X7R antagonist OxATP, they were blocked by the adenosine A_1_ receptor (A_1_R) antagonist 8-cyclopentyl-1,3-dipropylxanthine (DPCPX), suggesting possible BzATP hydrolysis to 2′(3′)-O-(4-benzoylbenzoyl)adenosine (Bz-adenosine) and consequent activation of A_1_Rs as a mechanism. Taken together, these data point out that 1) P2X7R expression and activity are confirmed to be altered in the presence of HD mutation; 2) in some experimental settings, such an abnormal functioning can be ascribed to presynaptic A_1_Rs activation.

## Introduction

Huntington's disease (HD) is a genetic neurodegenerative disease characterized by a complex set of movement, psychiatric, and cognitive alterations (Harper and Newcombe, 1992).

The disease is caused by a mutation in the exon 1 of the *IT-15* gene, located on chromosome 4, encoding for the huntingtin (htt) protein (The Huntington’s Disease Collaborative Research Group, 1993). The mutation is an expansion (above the threshold of 36 repetitions) of a CAG trinucleotide which encodes for a long poly-glutamine (poliQ) tract within the htt protein. Abnormal htt protein aggregates have been found at the neuronal level in both HD patients and experimental models (Reddy et al., 1999). Despite the ubiquitous expression of mutant and normal htt protein, the neurodegeneration mainly involves the striatum, with neuronal death selectively occurring in medium-sized spiny neurons (MSNs) and, to a lesser extent, in the cortex (Vonsattel et al., 1985). Since these brain areas represent the paradigmatic site of the corticostriatal glutamatergic pathway, a role for an altered glutamate receptor signaling, and consequent excitotoxicity, has been proposed to explain MSN vulnerability (Levine et al., 1999; Cepeda et al., 2001; Zeron et al., 2002; Martire et al., 2007).

Purinergic P2 receptor family comprises the P2Y G protein-coupled receptors and P2X receptors (P2X1–7), which are ATP-sensitive ligand-gated ion channels (Ralevic and Burnstock, 1998; Abbracchio et al., 2006). Among ionotropic P2X receptors, the P2X7 subtype (P2X7R) is one of the most promising targets for many pathological conditions of the central nervous system. Increasing evidence indicates that deregulated expression and activation of the P2X7R play a role in neurodegenerative disorders (Volonté et al., 2012; Tewari and Seth, 2015; Miras-Portugal et al., 2017; Francistiová et al., 2020). The receptor is expressed in a wide range of cell types in the brain (Yu et al., 2008), facilitates presynaptic neurotransmitter release (Sperlágh et al., 2002), and induces apoptosis in neurons (Nishida et al., 2012). Changes in P2X7R function and expression may contribute to HD pathogenesis, and P2X7R antagonists may have some therapeutic potential in HD; indeed, primary cultured neurons bearing the htt mutation are more susceptible to P2X7R-induced apoptosis, and *in vivo* administration of a P2X7R antagonist attenuates motor impairments in HD mice (Diaz-Hernandez et al., 2009). Very interestingly, P2X7R has been recently found altered in the brain of HD subjects (Ollà et al., 2020).

However, the nature of the P2X7R alteration in HD is still poorly explored, and the mechanisms responsible for P2X7R-mediated effects are largely unknown. In the present study, we evaluated the expression and the functioning of P2X7R in two genetic models of HD, different from those (Tet/HD94 and R6/1) previously investigated in Diaz-Hernandez et al. (2009). Namely, we used ST14A rat striatal cells, expressing full-length wild-type (WT, Q15) or mutant (Q120) htt (Cattaneo and Conti, 1998; Ehrlich et al., 2001), and R6/2 mice (Mangiarini et al., 1996), the most widely used transgenic model of HD, resembling juvenile forms of the disorder given the rapid development of symptoms. In the presence of HD mutation, we found an altered P2X7R expression and a larger P2X7R response to the agonist 2′(3′)-O-(4-benzoylbenzoyl)adenosine-5′-triphosphate (BzATP), which induced cell death and reduced synaptic transmission. Finally, in the electrophysiology experimental setting, we demonstrated that the BzATP effect observed in corticostriatal slices strongly depends on presynaptic A_1_Rs activation.

## Materials and Methods

### Cell Cultures

#### Q15 and Q120 ST14A Cell Cultures

ST14A/Q15 and ST14A/Q120 cell lines were provided by the Coriell Biological Material Repository by the High Q Foundation and CHDi, Inc. These cells express a human htt N-terminal portion (residues 1–548); ST14A/Q15 cells express normal htt with a 15-glutamine repeat region, while ST14A/Q120 cells express mutant htt with a 120-glutamine repeat region. The cells were developed from embryonic day 14 rat striatal primordia by retroviral transduction of the temperature-sensitive SV40 large T antigen (Cattaneo and Conti, 1998) and have typical features of MSNs that are affected in HD (Cattaneo and Conti, 1998; Ehrlich et al., 2001). The cells were grown at a permissive temperature of 33°C under 5% CO_2_ in high-glucose Dulbecco’s modified Eagle’s medium (DMEM) containing 10% fetal bovine serum (FBS), 2 mM L-glutamine, 100 units/mL penicillin, and 0.1 mg/ml streptomycin (P/S), hereafter referred to as the complete medium. Under these conditions, ST14A/Q15 and ST14A/Q120 cells grew similarly. DMEM, FBS, L-glutamine, and P/S were purchased from Euroclone.

#### Cells Treatment and Viability Measurement

Cells were plated in complete medium for 24 h which was replaced with medium without FBS for an additional 24 h. BzATP was added for 5 h in Locke’s medium that was then replaced with medium without FBS for 48 h. Periodate-oxidized ATP (OxATP) or 8-cyclopentyl-1,3-dipropylxanthine (DPCPX) was added 1 h before BzATP and kept throughout the treatment. All the experiments were performed at permissive temperature and in the absence of FBS to stop cell proliferation (Ehrlich et al., 2001). Trypan blue staining was used to evaluate cell death. Cells were trypsinized and incubated with 0.1% trypan blue. A hemocytometer was used to count unstained viable cells.

### Protein Extraction, SDS-PAGE, and Western Blotting

ST14A cells were cultured in 25 cm^2^ flasks and then collected in ice-cold RIPA buffer (PBS, 1% Nonidet P-40, 0.5% sodium deoxycholate, and 0.1% SDS) supplemented with protease inhibitor cocktail (Roche). Lysates were kept 1 h on ice and then centrifuged for 10 min at 15000 ×  g at 4°C. Supernatants were collected, and protein concentration was determined by Bio-Rad Protein Assay (Bio-Rad), based on the Bradford dye-binding method (Bradford, 1976), using bovine serum albumin (BSA) as a standard. Samples were boiled for 5 min for complete protein denaturation. Total protein samples (18 µg) were separated by 10% SDS-PAGE electrophoresis and then blotted onto nitrocellulose membranes (Protran, Amersham) in 20% ethanol Tris-Glycine buffer. Membranes were blocked in 5% non-fat dry milk TBS-T buffer (10 mM Tris pH 8, 150 mM NaCl, 0.1% Tween 20) and incubated overnight with rabbit anti-P2X7R (1:500, Bioss) or goat anti-Lamin B1 (1:1000, MyBioSource) primary antisera. HRP-conjugated anti-rabbit or anti-goat secondary antisera (1:5000 diluted in 5% non-fat dry milk TBS-T buffer) were incubated with membranes for 1 h at room temperature. Immunostained bands were visualized using ECL Select reagent (GE Healthcare) on iBright CL1000 Imaging System (Invitrogen) and analyzed using Image J software.

### Animals

All animal procedures were carried out according to the principles and procedures outlined in the European Community Guidelines for Animal Care, DL 26/2014, application of the European Communities Council Directive, 2010/63/EU, FELASA and ARRIVE guidelines. All animal procedures were approved by the Italian Ministry of Health and by the local Institutional Animal Care and Use Committee (IACUC) at Istituto Superiore di Sanità (Rome, Italy). A colony of R6/2 transgenic and WT littermate mice was maintained on the CBAxC57BL/6 hybrid background at Charles River Laboratories. Male and female genotyped mice, usually not younger than 4.5 weeks of age, were delivered and housed in our animal facilities until the end of the experiments. On their 12^th^ week of age, symptomatic R6/2 mice and WT littermates were sacrificed by cervical dislocation and used for electrophysiological experiments. The animals were kept in standard cages (48 × 26 × 20 cm, four mice per cage) under standardized temperature (22°C), humidity (55%), and lighting conditions (12:12 h light:dark cycle, with lights on at 6 am) with free access to water and food. For animals used in the present study, proper treatment, care, and humane conditions have been provided. All efforts were made to reduce the number of animals used and to minimize their pain and discomfort. Permanent veterinary surveillance and animal welfare evaluation have been provided by the Host Institution.

### Electrophysiology

#### Slice Preparation and Recordings

Corticostriatal slices were prepared according to the method described by Tebano et al. (2009). Transgenic R6/2 mice in a late symptomatic phase (12 weeks of age) and age-matched WT were used. Animals were sacrificed by cervical dislocation, the brain was removed from the skull, and coronal slices (300 µm thick) including the neostriatum and the neocortex were cut with a vibratome. Slices were maintained at 22–24°C in artificial cerebrospinal fluid (ACSF) containing (mM) 126 NaCl, 3.5 KCl, 1.2 NaH_2_PO_4_, 1.2 MgCl_2_, 2 CaCl_2_, 25 NaHCO_3_, and 11 glucose, saturated with 95% O_2_ and 5% CO_2_ (pH 7.3). After incubation in ACSF for at least 1 h, a single slice was transferred to a submerged recording chamber and continuously superfused at 32–33°C with ACSF at a rate of 2.6 ml/min. Tested drugs were added to this superfusion solution. Extracellular field potentials (FPs) were recorded in the dorsomedial striatum with a glass microelectrode filled with 2 M NaCl solution (pipette resistance 2–5 MΩ) on stimulation of the white matter between the cortex and the striatum with a bipolar twisted NiCr-insulated electrode (50 µm o.d.). Each pulse was delivered every 20 s with duration of 100 µs and an intensity chosen to cause 60% of maximal response. Three consecutive responses were recorded and averaged using a DAM-80 AC differential amplifier (WPI Instruments), acquired, and analyzed using LTP software (Anderson and Collingridge, 2001). At least 10 min of stable baseline recording preceded drug application. The data were expressed as mean ± SEM from n experiments (one slice tested in each experiment, slices were obtained from at least two animals for each set of the experiment). To allow comparisons between different experiments, the FP amplitudes were normalized in each experiment, taking as 100% the average of values obtained over the 10 min period immediately before the test compound was applied, and considered as the basal value. The effects of the drugs were expressed as a percentage variation of basal values over the last 5 min of drug perfusion.

#### Paired-Pulse Stimulation

In the PPS protocol, two consecutive pulses were applied 50 msec apart. In the control condition, this protocol elicits paired-pulse facilitation (PPF), a form of short-term plasticity triggered by presynaptic mechanisms, in which the response elicited by the second stimulus (R2) is greater than that elicited by the first stimulus (R1). The degree of PPF is quantified by the R2/R1 ratio.

### Drugs

BzATP (P2X7R agonist) and OxATP (P2X7R antagonist) were purchased from Sigma-Aldrich. DPCPX (A_1_R antagonist) was purchased from Tocris Biosciences. Pharmacological agents were dissolved in distilled water (BzATP, OxATP) or DMSO (DPCPX). In this last case, stock solutions were made to obtain concentrations of DMSO lower than 0.001% in the superfusing ACSF and cell culture media. This DMSO concentration did not affect basal synaptic transmission in corticostriatal slices (not shown).

### Statistics

Results from *in vitro* and western blotting experiments were expressed as a percentage of control, which was considered as 100%, and as mean ± SEM values of at least three independently performed experiments (each independent experiment corresponds to an independent cell culture preparation). Results from electrophysiology experiments were expressed as mean ± SEM from n slices. In the legends, the number of animals from which the slices have been obtained is reported for each data set. A *p* < 0.05 was considered to indicate a significant difference. Statistical analysis of the data was performed using Mann–Whitney test for *in vitro* and electrophysiology experiments and one-sample *t*-test for western blotting. Statistical analyses and curve fittings were obtained by using GraphPad Prism software (version 6.05; GraphPad Software).

## Results

### Evaluation of P2X7R Expression in Huntington’s Disease Cell Line

**FIGURE 1:**
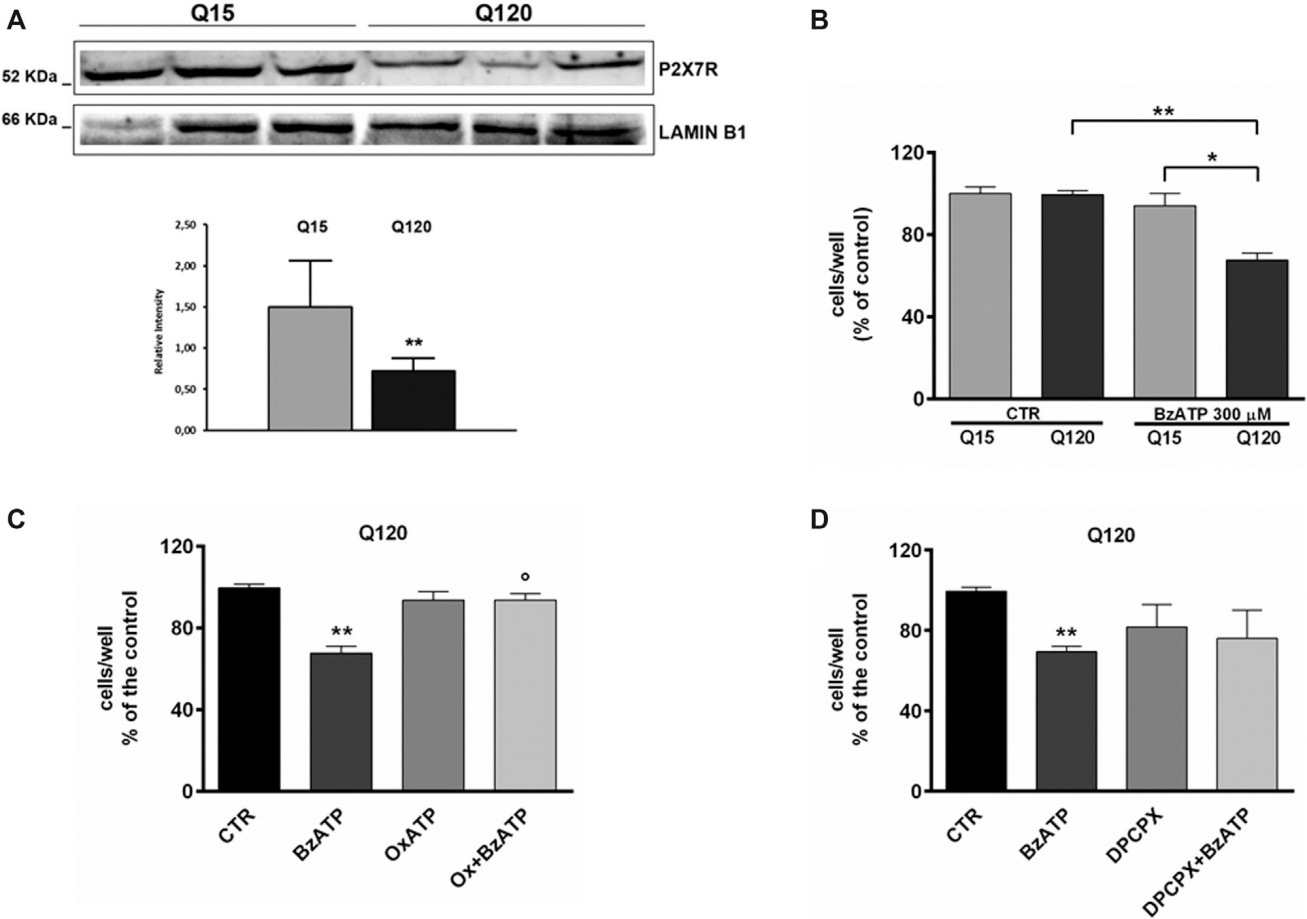
P2X7R expression is reduced in HD cells. **(A)** Western blot analysis in HD cells and relative control shows that P2X7R expression is reduced in presence of mutant htt. Lamin B1 is used as a loading control. The result is expressed as the mean ± SEM of four independent biological replicates (**p < 0.01 according to One-Sample *t*-test). BzATP effects in HD cell line. **(B)** The treatment with BzATP 300 μM was ineffective in ST14A/Q15 whereas it reduced cell viability in ST14A/Q120 (**p < 0.01 vs. Q120/CTR and *p < 0.05 vs. Q15/BzATP, according to Mann–Whitney test). **(C)** The BzATP effect in ST14A/Q120 (**p < 0.01 vs. Q120/CTR) was blocked by the P2X7R antagonist OxATP (1 μM; °*p* < 0.01 vs. BzATP-treated Q120 cells, according to Mann–Whitney test) but not by the A_1_R antagonist DPCPX **(D)**.

First of all, we verified the extent of P2X7R expression in our HD cell line by western blotting analysis. We found that while P2X7R is expressed in both genotypes, its level is significantly lower in mutant cells in comparison to WT cells (Figure 1, panel A).

### BzATP-induced toxicity in Q120 cells was blocked by the P2X7R antagonist but not by the A_1_R antagonist

It has been demonstrated that P2X7R stimulation increases susceptibility to cell death in neuronal cell lines and apoptosis in cultured neurons expressing mutant htt (Jun et al., 2007; Diaz-Hernandez et al., 2009). To verify whether the stimulation of P2X7R induced cellular death also in our *in vitro* model of HD, we treated ST14A/Q15 and ST14A/Q120 with the P2X7R agonist BzATP.

Preliminarily, the concentration of BzATP 300 µM was selected based on a concentration-effect experiment (data not shown). At this concentration, BzATP induced a significant toxic effect in Q120 cells (67.67 ± 3.43% of control, *n* = 6; *p < 0.05 vs. Q15 and **p < 0.01 vs. CTR, according to Mann–Whitney test; Figure 1, panel B) but not in Q15 cells.

BzATP-induced toxicity in Q120 cells was completely blocked by pretreatment with OxATP 1 µM (93.67 ± 3.18% of control, *n* = 3; *p < 0.05 vs. BzATP alone, according to Mann–Whitney test; Figure 1, panel C). Given that extracellular catabolism of BzATP to 2′ (3′)-O-(4-benzoylbenzoyl)adenosine (Bz-adenosine), which in turn is hetero-exchanged for intracellular adenosine, can occur (Ireland et al., 2004; Kukley et al., 2004) and it has been previously demonstrated that activation of A_1_R can exert a pro-toxic effect in HD cells (Ferrante et al., 2014), we decided to rule out the involvement of A_1_Rs by using the specific antagonist DPCPX. In Q120 cells, DPCPX (100 nM) did not significantly reduce BzATP toxicity (76.00 ± 14.15% of control, n = 3; *p* = 0.82 vs. BzATP alone, according to Mann–Whitney test; Figure 1, panel D).

### BzATP-Induced Depression of Synaptic Transmission is Larger in R6/2 Mice

**FIGURE 2:**
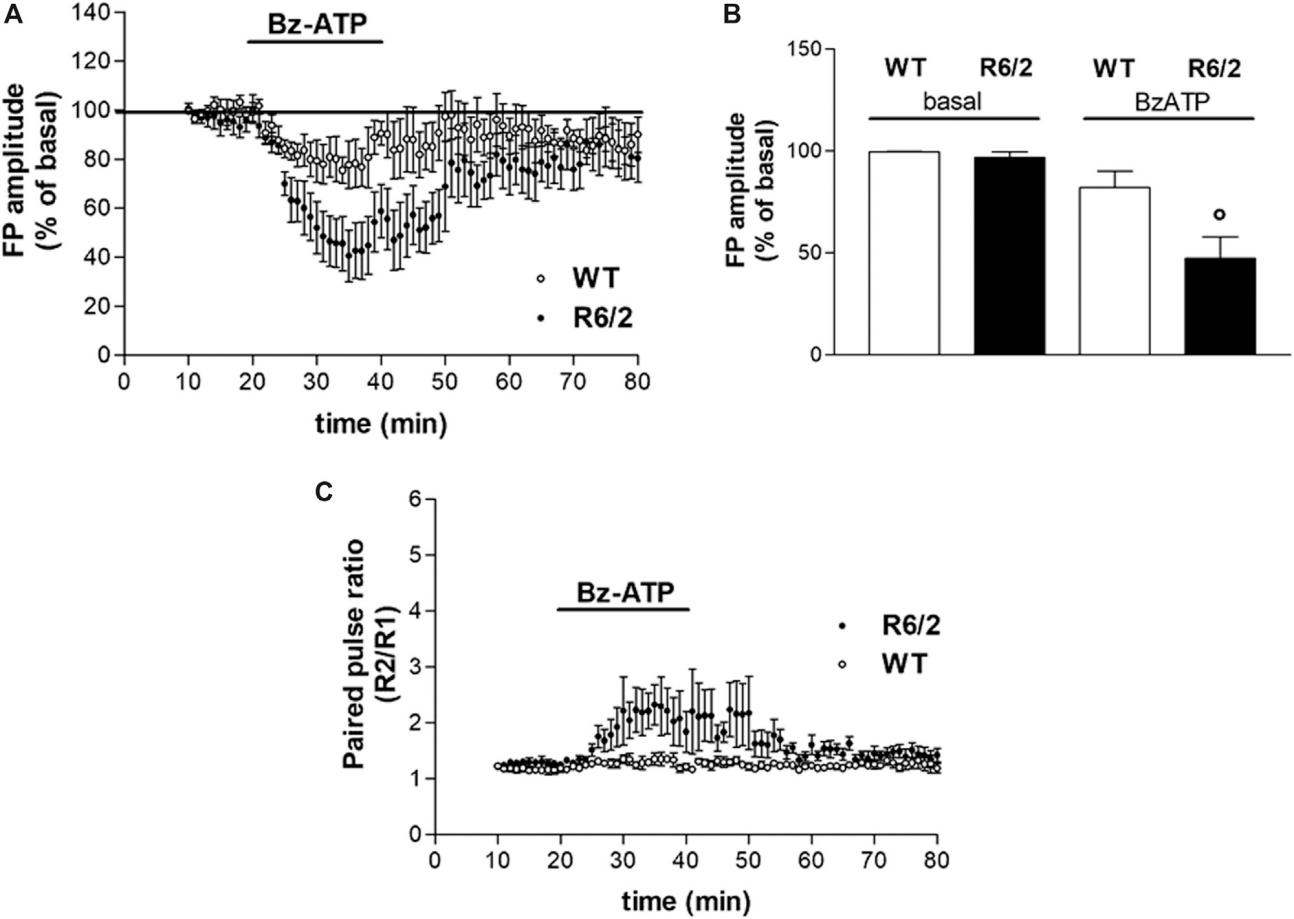
BzATP effects on synaptic transmission in corticostriatal slices from WT and R6/2 mice. **(A)** BzATP 50 μM induced a larger depression of field potential (FP) amplitude in R6/2 than in WT mice. The horizontal bars indicate the period of drug application. **(B)** The histograms show the mean ± SEM of the FP amplitude expressed as a mean percentage variation of baseline during the last 5 min of drug perfusion. BzATP effect was significantly larger in R6/2 mice; °*p* < 0.05 vs. WT and vs. basal, according to Mann–Whitney test. **(C)** Effect of BzATP on presynaptic neurotransmitter release. While basal R2/R1 ratio was comparable, after BzATP application it was significantly higher in R6/2 compared to WT mice (2.13 ± 0.39%, *n* = 6 and 1.29 ± 0.08%, respectively).

We confirmed that R6/2 mice resulted comparable to WT mice in terms of corticostriatal basal synaptic transmission, as previously reported (Martire et al., 2013). In extracellular electrophysiology experiments, R6/2 and littermate WT mice were used. The application of BzATP (50 μM for 20 min) induced a reduction of FP amplitude in both WT and R6/2 corticostriatal slices. However, BzATP-induced reduction of FP was statistically significant in transgenic but not in WT mice (47.27 ± 10.61%, *n* = 6, and 82.08 ± 8.12%, *n* = 4, respectively; °*p* < 0.05 vs. WT and vs. basal, according to Mann–Whitney test; Figure 2, panels A and B). The concentration of BzATP (50 μM) was chosen after a 30 μM trial resulted ineffective in WT slices (not shown).

### BzATP Induced a Larger Inhibition of Presynaptic Glutamate Release in R6/2 Mice

Several lines of evidence suggest that activation of P2X7R can increase the release of glutamate from neural tissues, such as hippocampal slices, isolated synaptosomes, and brainstem slices (Lundy et al., 2002; Sperlágh et al., 2002; Ireland et al., 2004). According to the reported stimulatory action of the P2X7R agonist BzATP on neurotransmitter release, we explored whether the BzATP effects on FP amplitude were mediated by a presynaptic mechanism. As presynaptic effects are expected to be accompanied by changes in the paired-pulse ratio (PPR; Schulz et al., 1994), we analyzed the FP amplitude ratios of paired pulses delivered with a 50 ms interval. Contrary to what was expected, all along with the BzATP treatment and immediately after, we found a BzATP-induced increase of the mean PPR, which rather indicates a reduction of presynaptic neurotransmitter release. Furthermore, this effect was larger in slices obtained from R6/2 mice than in those of WT animals (2.13 ± 0.39%, *n* = 6 and 1.29 ± 0.08%, *n* = 4, respectively; Figure 2C).

### BzATP-Induced Depression of Corticostriatal Synaptic Transmission Is Reversed by an A_1_R Antagonist

**FIGURE 3:**
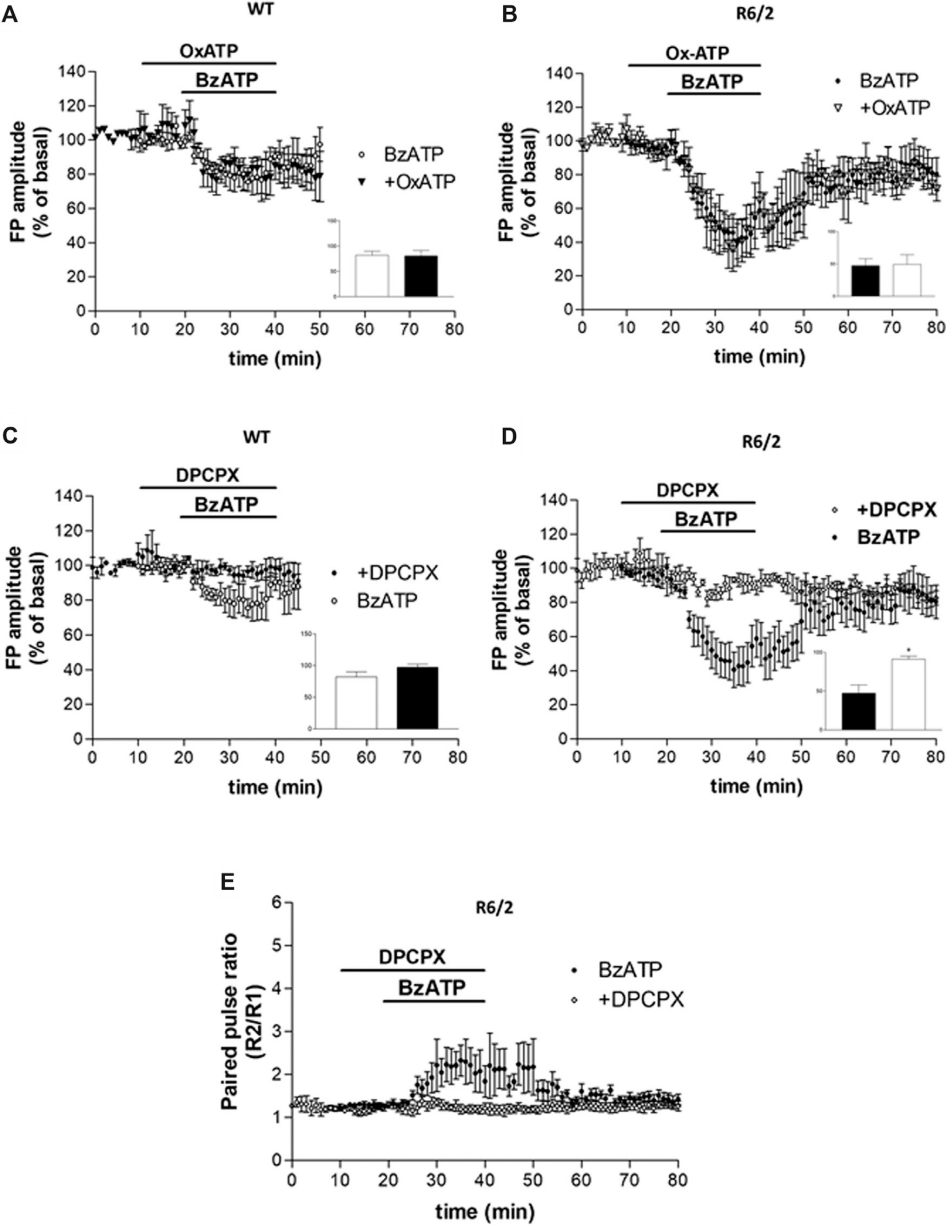
**(A,B)** BzATP inhibitory effect was not prevented by the P2X7R antagonist OxATP, in neither WT nor R6/2 mice (insets in panels **A,B**). **(C,D)** The BzATP-induced depression was reduced in WT and completely abolished in R6/2 slices if DPCPX was pre-applied (°*p* < 0.01 vs. BzATP, according to Mann–Whitney test; inset in panel **D**). **(E)** In R6/2 slices, the increase of PPR caused by BzATP was completely abolished by DPCPX.

While we first assumed that the BzATP effect on corticostriatal synaptic transmission was caused by the activation of P2X7Rs, after the PPS evaluation we speculated that some other presynaptic inhibitory receptors could be involved. In agreement with such a hypothesis, the inhibitory effect of BzATP was not prevented by the P2X7R antagonist OxATP (10 μM, for 30 min, Figure 3, panels A and B) neither in WT nor in R6/2 mice (80.63 ± 10.69%, *n* = 3 and 49.18 ± 15.06%, *n* = 3, respectively; insets in panels A and B of Figure 3). When given alone, OxATP was devoid of effect in both genotypes (not shown). However, since corticostriatal transmission and striatal glutamate outflow in R6/2 and WT mice are differently affected by the A_1_R agonist N^6^-cyclopentyladenosine (CPA) via presynaptic inhibitory A_1_Rs (Ferrante et al., 2014), and considering that ectonucleotidases in brain slices can potently and rapidly convert ATP and many of its analogs to adenosine (Funk et al., 1997; Zimmermann, 1996; Dunwiddie et al., 1997; Cunha et al., 1998; Kukley et al., 2004), we wondered whether the synaptic depression induced by BzATP could be blocked by the A_1_R antagonist. This was indeed the case since the BzATP-induced depression was reduced in WT and completely abolished in R6/2 slices if DPCPX (500 nM, for 30 min; Figure 3, panels C and D) was pre-applied (97.33 ± 4.88%, *n* = 3 and 91.32 ± 3.73%, *n* = 5, respectively; °*p* < 0.01 vs. BzATP, according to Mann–Whitney test; insets in panels C and D of Figure 3). Consequently, in R6/2 slices, the increase of PPR caused by BzATP was fully abolished by DPCPX (Figure 3, panel E). At this concentration, when given alone DPCPX did not significantly affect basal synaptic transmission (not shown).

## Discussion

In the present work, we evaluated the expression and functioning of P2X7R in HD and validated some results previously reported in different genetic models (Diaz-Hernandez et al., 2009). Our main results can be summarized as follows: 1) larger toxicity of P2X7R was found in ST14/Q120 cells, a rat striatal cellular model of HD, in comparison to WT cells; 2) a larger inhibitory effect of BzATP on synaptic transmission was also observed in the striatum of symptomatic R6/2 mice in comparison to WT mice; 3) while the BzATP-mediated toxic effects resulted to be P2X7R-dependent, its ability to induce synaptic depression appeared to be A_1_R-dependent.

By investigating the mechanism of the increased toxicity elicited by BzATP in our HD ST14A/Q120 cell culture model, we firstly excluded an increased expression of the receptor. Conversely, our western blotting studies revealed a significant downregulation of P2X7R in the presence of mutant htt. Although the reduction in receptor density might be considered a failed cellular strategy to prevent toxicity, in the presence of mutant htt, P2X7R stimulation can elicit pro-toxic effects that were potentiated in comparison to the normal htt condition. This finding is not the first one concerning the huge functional changes that receptors may deal with in the presence of mutant htt. A lack of linearity between the levels of P2X7R and the intensity of its effects is indeed not surprising, and a similar behavior has been previously reported, for instance, for adenosine A_2A_ receptors (A_2A_Rs). Although a marked reduction in the expression of A_2A_Rs has been found in HD patients (Glass et al., 2000) and in HD mice (Cha et al., 1999), a normal or even higher response of these receptors was also observed in R6/2 mice (Varani et al., 2001; Chou et al., 2005; Tarditi et al., 2006).

In agreement with the known ability of P2X7R to mediate apoptosis consequently to elevations of intracellular calcium in neurons (Jun et al., 2007), we do not exclude this mechanism in the toxicity caused by BzATP in HD ST14A/Q120 cells. Further experiments will verify this hypothesis.

Since in some experimental settings BzATP is supposed to undergo extracellular hydrolysis to Bz-adenosine, which in turn is hetero-exchanged for intracellular adenosine that finally activates A_1_Rs (Ireland et al., 2004; Kukley et al., 2004), we verified the involvement of the A_1_R stimulation.

*In vitro*, the A_1_R antagonist DPCPX did not block BzATP-induced toxicity in ST14A/Q120 cells, while such an effect was counteracted by the P2X7R antagonist OxATP, thus confirming a direct involvement of P2X7Rs.

In extracellular electrophysiology settings, we found that BzATP induced a larger depressive effect on synaptic transmission in the striatum of symptomatic R6/2 mice. Although P2X7R activation is generally known to enhance excitatory neurotransmitter release (Lundy et al., 2002; Sperlágh et al., 2002; Ireland et al., 2004), this effect was likely due to increased inhibitory action of BzATP on glutamate release, as suggested by the increase in PPR observed during the recordings in R6/2 slices. The BzATP-mediated synaptic depression was unresponsive to P2X7R antagonist OxATP while being completely blocked by the A_1_R antagonist DPCPX, an effect mirrored in terms of PPR. Even though we cannot completely rule out a direct effect of BzATP on P2X7R, given the long lag phase (∼5 min) of BzATP-induced synaptic depression, we can reasonably presume that its targeted receptor is unlikely to be P2X7R. Moreover, it must be taken into account that BzATP can activate all P2X subtypes characterized (Jarvis and Khakh, 2009), by acting also as a ligand of P2Y1, P2Y11, and P2Y12 receptors (Abbracchio et al., 2006). Finally, as an alternative possibility, BzATP hydrolysis to adenosine by ectonucleotidases could occur in striatal slices, similarly to what was previously observed in both the brainstem (Ireland et al., 2004) and hippocampal (Kukley et al., 2004) slices, with A_1_R activation significantly contributing to the BzATP-mediated FP inhibition and PPR increase. This would be consistent with the notion that A_1_Rs mediate adenosinergic inhibition at many central synapses (Dunwiddie, 1985).

So far, we can hypothesize that the lack of a comparable involvement of the A_1_R in ST14A/Q120 cells might be due to a reduced process of BzATP catabolism *in vitro* or to the absence of a clear pre-post synaptic compartmentalization in this experimental paradigm.

We are aware that future investigations will better characterize the *ex vivo* effects of BzATP since non-selective and off-target actions of OxATP have been previously reported (Beigi et al., 2003, and references therein). For instance, OxATP could be tested at higher concentrations or for longer incubation time than we did (10 μM, for 30 min in corticostriatal slices), or the more selective and potent P2X7R antagonist A-740003 (IC50 = 18 nM for rat receptor, Honore et al., 2006; Neves et al., 2020) could be used.

As detailed in the Supplements, the P2X7R antagonist, namely, Coomassie Brilliant Blue G (BBG), tested in corticostriatal slices against BzATP in WT mice and NMDA toxicity in both genotypes, did not induce any significant effect (Supplementary Figures S1, S2). Of course, the possibility that functional P2X7R could not be correctly expressed in the murine HD model used here should be also considered.

As elegantly demonstrated by Kukley et al. (2004) in hippocampal mossy fibers, BzATP, once catabolized to Bz-adenosine at the extracellular level and consequently hetero-exchanged for adenosine intracellularly, was able to depress excitatory post-synaptic potentials through presynaptic A_1_Rs rather than through P2X7Rs. BzATP effects are blocked by inhibitors of the enzymes involved in the ATP catabolism to adenosine, adenosine deaminase (ADA), and by blocking nucleoside transporters (NTrans). According to this evidence and the model proposed by Kukley et al., Bz-adenosine, which should be generated from the catabolism of BzATP, does not likely activate A_1_Rs given the benzoyl–benzoyl group at C3 of the ribose (Klotz, 2000) and cannot be metabolized in the extracellular space (given the absence of esterase activity in this compartment, Satoh and Hosokawa, 1998), but it should be transported intracellularly via NTrans and converted to adenosine, which, once released, finally activates A_1_Rs (Kukley et al., 2004).

The role of the activation of A_1_Rs in inhibiting glutamatergic transmission in corticostriatal slices is well known (Calabresi et al., 1997). Moreover, we previously demonstrated an increased sensitivity to the A_1_R agonist CPA both in the HD striatal cell line STHdh111/111 and in the R6/2 mice, being the CPA inhibitory effect on the striatal synaptic transmission (similarly to what obtained with BzATP as shown here) more pronounced in HD than in littermate WT animals, also in terms of presynaptic glutamate release inhibition (Ferrante et al., 2014). Besides, we found lower levels of ADA in the cytosol of R6/2 mice (Ferrante et al., 2014), this reduction maybe contributing to the higher extracellular adenosine concentration reported in the striatum of symptomatic R6/2 mice (Gianfriddo et al., 2004). This set of data further supported our present results.

Far from being conclusive, we report here that HD mutation induces substantial changes in P2X7Rs expression, pharmacology, and functions and that, in some experimental settings, such an abnormal functioning can be ascribed to presynaptic A_1_Rs activation, which is similarly and abnormally augmented in HD genetic models (Ferrante et al., 2014). The issue regarding the poor stability of BzATP and the caution required in using such a P2X7R agonist have to be taken into due account, particularly when performing electrophysiology experiment (Ireland et al., 2004; Kukley et al., 2004) and, more in general, in the preclinical assessment of P2X7R as a potential therapeutic target to treat HD or other neurodegenerative disorders. However, the nature of the P2X7R alteration in HD deserves future explorations, since the mechanisms responsible for P2X7R-mediated effects are still largely unknown. The identification of such mechanisms is thus essential to establish the actual therapeutic potential of P2X7 antagonists in HD and becomes topical since, very recently, P2X7R has been found altered in the brain of HD subjects, in terms of increased transcription/expression, and abnormal gene splicing (Ollà et al., 2020).

## Data Availability

The raw data supporting the conclusions of this article will be made available by the authors, without undue reservation.
