# Supplementary material for: P2X7 Receptor Agonist 2′(3′)-O-(4-Benzoylbenzoyl)ATP Differently Modulates Cell Viability and Corticostriatal Synaptic Transmission in Experimental Models of Huntington’s Disease
Source: Front Pharmacol. 2021 Feb 19;11:633861. doi: 10.3389/fphar.2020.633861 (PMC7933594; doi:10.3389/fphar.2020.633861)
Supplement: Supplementary file 1 [file datasheet1.pdf]

Frontiers in Pharmacology - Supplementary Information

Martire et al.

Supplemental Figures

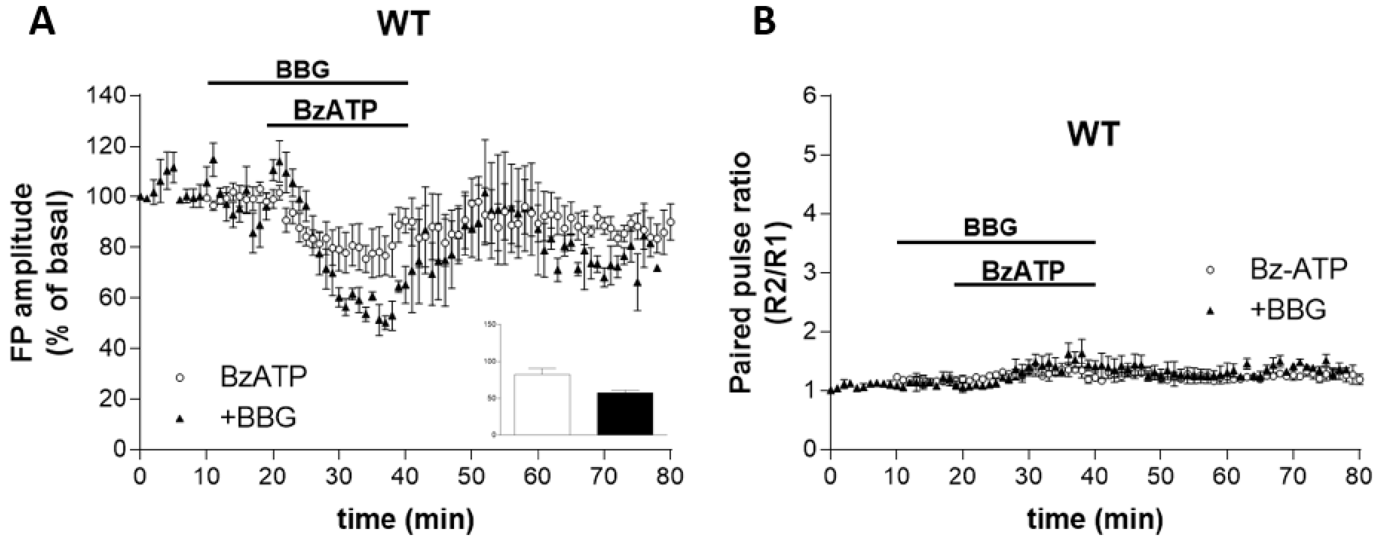

Figure S1.

BzATP-induced depression of corticostriatal synaptic transmission in WT mice is not reversed by the P2X7R antagonist Coomassie Brilliant Blue G (BBG)

In WT mice, the inhibitory effect of BzATP (50 µM for 20 min) on FP (82.08 ± 8.12% of basal, n= 4) was not prevented by the P2X7R antagonist BBG (1 µM, for 30 min) (57.42 ± 3.43%, of basal n = 3, panel A). On the contrary, a slight and non-significant worsening was induced. The trend of the mean PPR resulted superimposable with or without BBG (panel B), indicating a substancial absence of any effect. According to Jiang et al. (2000), BBG 1 µM is below the IC(50) values for inhibition of the other P2XRs, thus suggesting P2X7R selectivity. BBG (Sigma-Aldrich) was dissolved in distilled water.

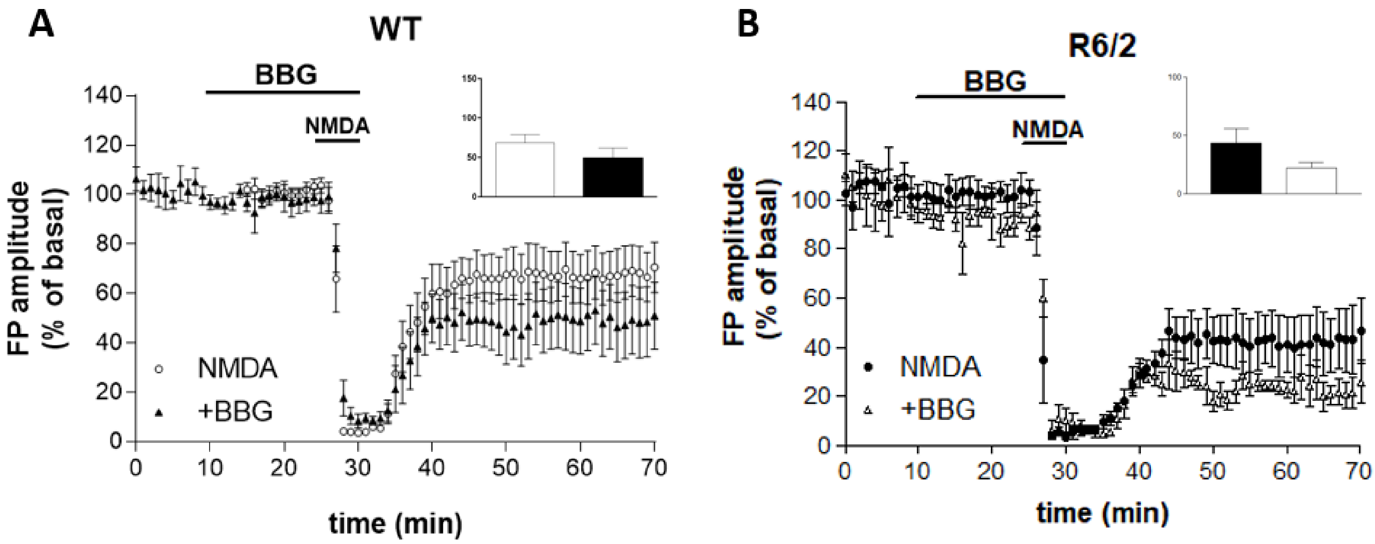

Figure S2.

P2X7R antagonist BBG did not reduce NMDA toxicity in corticostriatal slices from R6/2 and WT mice

The NMDA experimental paradigm has been already used by our group to assess the role of A_{2A} (Martire et al., 2007) and A_1 (Ferrante et al., 2014) receptors in excitotoxicity in corticostriatal slices from WT and R6/2 mice. NMDA (75 µM) is applied to the slices over 5 min, inducing a reduction of FP amplitude which results increased in R6/2 mice. In WT mice (panel A) the recovery of the FP after NMDA application (68.31 ± 10.44% of basal after 40 min of washout, n = 7) was not significantly affected by the BBG treatment (1 µM, for 20 min) (48.32 ± 13.80% of basal after 40 min of washout, n = 7). In R6/2 mice (panel B) NMDA toxicity was higher (43.20 ± 12.68% of basal after 40 min of washout, n = 3) than in WT mice, but also in this case BBG did not exert a protective action; on the contrary, a worsening, even if non-significant, was induced (21.69 ± 5.36% of basal after 40 min of washout, n = 4). NMDA (Sigma-Aldrich) was dissolved in NaOH 0.1 N.

References

Ferrante, A., Martire, A., Pepponi, R., Varani, K., Vincenzi, F., Ferraro, L., et al. (2014). Expression, pharmacology and functional activity of adenosine A1 receptors in genetic models of Huntington's disease. Neurobiol. Dis. 71, 193–204. doi:10.1016/j.nbd.2014.08.013.

Jiang, L.H., Mackenzie, A.B., North, R.A., Surprenant, A. (2000). Brilliant blue G selectively blocks ATP-gated rat P2X(7) receptors. *Mol. Pharmacol.* 58, 82-88. PMID: 10860929.

Martire, A., Calamandrei, G., Felici, F., Scattoni, M. L., Lastoria, G., Domenici, M. R., et al. (2007). Opposite effects of the A2A receptor agonist CGS21680 in the striatum of Huntington's disease versus wild-type mice. Neurosci. Lett. 417, 78–83. doi:S0304-3940(07)00189-9 [pii].
